# Supplementary material for: Na+ Translocation Dominates over H+-Translocation in the Membrane Pyrophosphatase with Dual Transport Specificity
Source: Int J Mol Sci. 2024 Nov 7;25(22):11963. doi: 10.3390/ijms252211963 (PMC11593465; doi:10.3390/ijms252211963)
Supplement: Supplementary file 1 [file ijms-25-11963-s001.zip › ijms-3269295-supplementary.pdf]

Supplementary Materials

Na^+ translocation dominates over H^+-translocation in the membrane pyrophosphatase with dual transport specificity

Alexander V. Bogachev, Viktor A. Anashkin, Yulia V. Bertsova, Elena G. Zavyalova, and Alexander A. Baykov

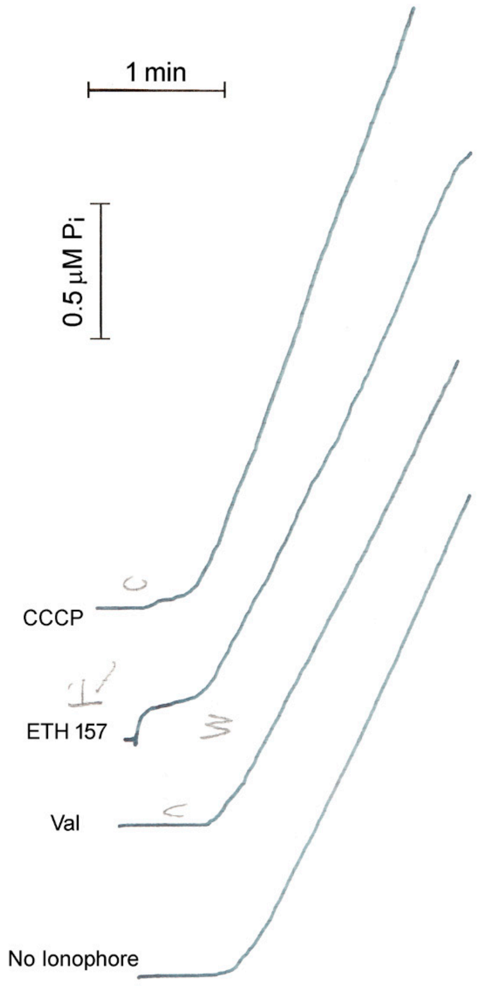

Figure S1. Effects of the ionophores on P_i production during PP_i hydrolysis by the membrane vesicles harboring *B. vulgatus* Na^+,H^+-translocating mPPase, as measured using a continuous P_i assay. The assay conditions are described in section 4.3, vesicle concentration was 2 μg protein/mL. Ionophore concentrations were as follows: valinomycin, 1 μM; ETH 157, 20 μM; CCCP, 10 μM.

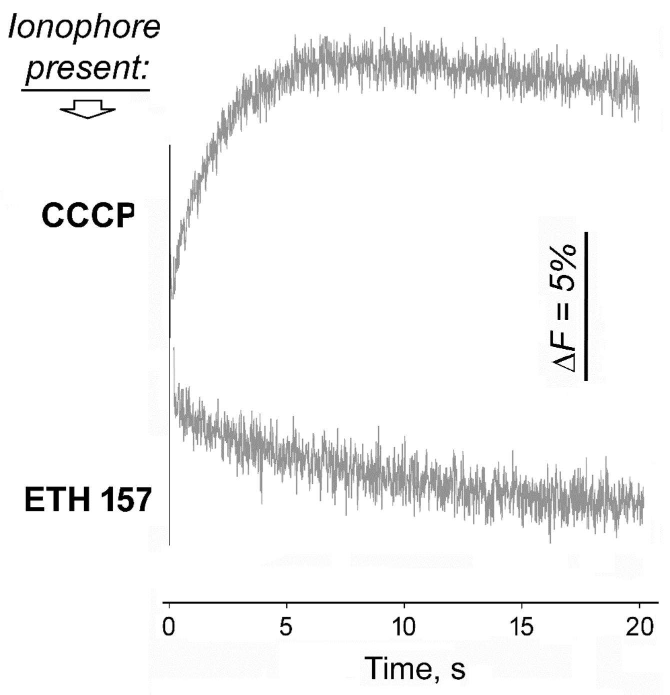

Figure S2. Stopped-flow traces of pyranine fluorescence recorded after adding 10 μM PP_i (final concentration) to the suspension of the membrane vesicles harboring *B. vulgatus* Na^+,H^+-translocating mPPase. Final Na^+ concentration was 10 mM. Other details of the assay were as described in Figure 5 legend.

Table S1. The effect of alamethicin on the NADH oxidase activity of the *Bv*-mPPase-containing membrane vesicles (data from a typical experiment)[a]

NADH oxidation rate (μmol/min per 1 mg protein)		Ratio (%)
- Alamethicin	+Alamethicin (17 μg/mL)	
0.61 ± 0.03	0.65 ± 0.04	94 ± 2

[a] Rates of NADH oxidation at 25°C were measured using a Hitachi 557 spectrophotometer at 340 nm (ε_{340} = 6.22 $mM^{-1}\cdot cm^{-1}$). The reaction mixture contained 100 mM Mops-KOH buffer, pH 7.2, 5 mM $MgSO_4$, 25 mM K_2SO_4, 5 mM Na_2SO_4, 2 μg/mL gramicidin D, and the membrane vesicles (10 μg protein/mL). All rate measurements were performed in duplicate. Alamethicin makes the membrane permeable to NADH. The rate measured without alamethicin refers to inverted ("inside out") membrane vesicles, whose NADH oxidase is exposed to solution. The ratio of the rates measured in the absence and presence of alamethicin is therefore the fraction of the inverted membrane vesicles in the whole population.

Table S2. Parameters of Equations 1 and 2, derived from stopped-flow data (Figures 5 and S2), as functions of Na^+ and PP_i concentrations. PP_i concentration was 150 μM, except where otherwise noted

[Na^+], mM	+ CCCP		+ ETH 157		v_0^{ETH}/v_0^{CCCP}
	v_0, %·s^{-1}	k, s^{-1}	v_0, %·s^{-1}	k, s^{-1}	
1	4.3 ± 0.1	0.52 ± 0.01	0.70 ± 0.04	0.083 ± 0.003	0.16 ± 0.01
10	6.5 ± 0.2	0.64 ± 0.02	0.84 ± 0.07	0.12 ± 0.01	0.13 ± 0.02
10 (10 μM PP_i)	5.0 ± 0.1	0.58 ± 0.01	0.60 ± 0.04	0.115 ± 0.005	0.12 ± 0.01
50	9.8 ± 0.4	0.89 ± 0.02	1.68 ± 0.07	0.28 ± 0.01	0.17 ± 0.02
